# Correction: Mathematical Model-Driven Deep Learning Enables Personalized Adaptive Therapy

**DOI:** 10.1158/0008-5472.CAN-24-4438

**Published:** 2025-01-15

**Authors:** Kit Gallagher, Maximilian A.R. Strobl, Derek S. Park, Fabian C. Spoendlin, Robert A. Gatenby, Philip K. Maini, Alexander R.A. Anderson

In the original version of this article (1), there was a typographical error in Figs. 2B, 3A, 5B, 6B, Supplementary Fig. S1A, and Supplementary Fig. S3 where the labels “Resistant Fraction” and “Sensitive Fraction” were inadvertently switched. These errors have been corrected in the latest online HTML and PDF versions of the article. The authors regret these errors.
